# Neutrophil Chemotaxis in Cord Blood of Term and Preterm Neonates Is Reduced in Preterm Neonates and Influenced by the Mode of Delivery and Anaesthesia

**DOI:** 10.1371/journal.pone.0120341

**Published:** 2015-04-13

**Authors:** Alexandra Birle, C. Thomas Nebe, Sandra Hill, Karin Hartmann, Johannes Poeschl, Lutz Koch

**Affiliations:** 1 Department of Neonatology, University Children’s Hospital, Medical School, Heidelberg, Germany; 2 Hämatologie-Labor Mannheim, Mannheim, Germany; 3 Department of Neonatology, Catholic Children's Hospital Wilhelmstift, Hamburg, Germany; Case Western Reserve University, UNITED STATES

## Abstract

Bacterial infections, even without any perinatal risk factors, are common in newborns, especially in preterm neonates. The aim of this study was to evaluate possible impairment of neutrophil chemotaxis in term and preterm neonates compared with adults as well as neonates with different modes of delivery and anaesthesia. We analysed the expression of the adhesion molecule L-Selectin as well as shape change, spontaneous and N-formyl-methionyl-leucyl-phenylalanine (fMLP)-induced transmigration of neutrophils in a flow cytometric assay of chemotaxis after spontaneous delivery with Cesarian Section (CS) under spinal anaesthesia (mepivacaine, sufentanil), epidural anaesthesia (ropivacaine or bupivacaine, sufentanil) or general anaesthesia (ketamine, thiopental, succinylcholine). Chemokinesis was higher (p=0.008) in cord blood neutrophils than in the adult ones, whereas those could be more stimulated by fMLP (p=0.02). After vaginal delivery neutrophils showed a higher spontaneous and fMLP-stimulated chemotactic response compared to neonates after CS without labor. Comparing different types of anaesthesia for CS, spinal anaesthesia resulted in less impairment on chemotaxis than general anaesthesia or epidural anaesthesia. The new flow cytometric assay of neutrophil chemotaxis is an appropriate and objective method to analyse functional differences even in very small volumes of blood, essential in neonatology. Term neonates do not show reduced chemotaxis compared to adults. Preterm neonates present with reduced chemotaxis and chemokinesis, confirming the well known deficits in their neutrophil function. The side effects of maternal drugs on the neonatal immune system have to be considered especially when the immune response is already impaired, as in preterm infants.

## Introduction

Immunodeficiency is still one of the challenges in neonatology. Because innate immunology is mediated mainly by neutrophils those were always in the focus to explain increased risks of bacterial infections [1–5] beside monocytes [6]. They may be influenced by the mode of delivery and medical interventions like corticosteroids [7] as we discovered for nitric oxide inhalation and oxidative burst of neutrophils in newborns suffering from persistent pulmonary hypertension of the newborn (PPNH) [6]. Little is known about the clinical aspects of neutrophil adhesion and chemotaxis towards a gradient of chemotactic factors in inflammatory sites which are the initiating steps in the innate, unspecific immune response despite the fact that those cells play a key role in the immune response of the neonate, as the lymphatic system is not yet primed.

After rolling and adhesion on the vessel wall in the inflamed tissues the neutrophilic granulocyte becomes activated, changes its cellular shape, enlarges and sheds L-Selectin (CD62) together with other cell-surface adhesion molecules (L-Selectin-Shedding) [8,9]. The activated neutrophil squeezes through the endothelial cell layer and invades the tissue, where it is directed to the infection site by a number of endogenous and microbial chemoattractants. The terminal sequence of one of the constituents of the cell wall of Gram-negative bacteria, N-Formylmethionyl-Lencyl-Phenylalanin (fMLP) is one of the most potent stimulants of neutrophil chemotaxis. In the process of phagocytosis, the neutrophil engulfs and destroys the bacteria when the phagosome fuses with the lysosome by oxidative burst and proteolytic degradation that finally kills the neutrophil itself [10,11].

The aim of this study was to re-evaluate the role of the physiological process of neonatal neutrophil chemotaxis in cord blood (CB) cells by a newly developed objective and reproducible functional assay using a flow cytometric technique. Conventional assays for chemotaxis are variations of the classical Boyden chamber test. The Boyden chamber is a block made from plastic with defined holes in it. Each hole can be separated into an upper and lower compartment. These two compartments are created by introducing a filter membrane (disc) that is fixed by a screw with a central opening. The chemoattractant is filled in the lower part; the disc is layed in and fixed. Afterwards, the upper compartment is filled with the cell suspension. The forces that apply are sedimentation by gravity plus counterflow from the lower chamber.

Parameters to be taken for evaluation of the results are the number of cells in the lower compartment or the number of cells at the lower side of the filter membrane or the distance of the migration front from the upper side measured also after staining and microscopical observation. The limitations of these manual observations are subjective measurement of numbers and distances of cells and require high blood volumes for isolation and separation of neutrophils, which are limited in early and newborn infants.

In contrast, our new test uses pores with precise holes of 3 μm diameter (nucleopore) like cell culture inserts in 24 well cell culture plates. The cells from the lower part are removed by pipetting and transferred into a FACS tube for counting (adding known amounts of counting beads), analysis of shape change (light scatter) and loss of L-selectin (immunofluorescence).

Several deficits in their neutrophil function are well recognized [12], but little is known about their ability to migrate using a functional assay some of which were prone to artefacts e.g. of non-migrating granulocytes [13]. Neonatal neutrophil chemotaxis was suspected to be impaired after Caesarean Section (CS) due to the lack of the physiological process of labor [14–17], in preterms [18,19] and the use of local anaesthetic drugs [20–24]. Therefore we investigated neonates after CS under different types of anaesthesia and can present data of CB after maternal spinal anaesthesia, epidural anaesthesia and general anaesthesia.

## Methods

### Study population

Our cohort consisted of 44 term neonates (≥ 37 weeks of gestation) and 22 preterm neonates (< 37 weeks of gestation) without signs or maternal risk factors for neonatal infection, and of 30 adult volunteers. Children were born in the Department of Obstetrics and Gynaecology and treated in cooperation with the Department of Neonatology, University Children's Hospital, Heidelberg. Neonates with life-threatening malformations were excluded. For further characterization of the studied population see Tables 1 and 2. Blood sampling was performed in accordance with the principles of the Declaration of Helsinki. The local Ethics Committee at the University Hospital of Heidelberg, Germany approved the study, and all participants gave their written informed consent to the study.

**Table 1:** Demoscopic data and results of adults and term neonates.

	Adults	Term neonates				
Results (Mean ± SE)		Vaginal delivery	C.S. all types of anaesthesia	C.S. spinal anaesthesia	C.S. Epidural anaesthesia	C.S. general anaesthesia
**n**	30	13	31	13	11	7
**Gestational age (w)**		40 ± 1	38 ± 4	38 ± 1	38 ± 5	38 ± 5
**Birth weight (g)**		3737 ± 124	3176 ± 94	3065 ± 151	3118 ± 154	3456 ± 175
**Leukocytes/nl**	6.41 ± 0.24	12.57 ± 1.41	9.16 ± 0.73	11.1 ± 0.95	6.32 ± 0.76	9.73 ± 1.83
**Neutrophils/nl**	3.64 ± 0.21	6.75 ± 0.91	4.37 ± 0.51	5.77 ± 0.81	2.68 ± 0.71	4.18 ± 0.81
**Number of migrated PMN**						
**PBS**	1220 ± 251	5328 ± 1575	3134 ± 773	4845 ± 1532	2262 ± 888	1206 ± 509
**fMLP**	13495 ± 1456	9530 ± 1839	4574 ± 733	6100 ± 1184	3091 ± 1146	4068 ± 1385
**FSC**						
**PBS Insert**	375 ± 12.1	516.7 ± 14.6	441.5 ± 8.6	429.2 ± 12	442.5 ± 16.1	462.4 ± 17
**PBS**	404.6 ± 9.4	556.5 ± 11.8	481.2 ± 12.3	483.6 ± 9.3	455.9 ± 27.7	513 ± 29.4
**fMLP**	635.1 ± 10.4	622.1 ± 8.3	535.6 ± 14.6	553.8 ± 14.2	493.6 ± 32	567.6 ± 23.5
**L-Selectin (RFI)**						
**PBS Insert**	68.5 ± 3.3	22.4 ± 4	35.7 ± 3.7	47.6 ± 3.9	25 ± 6.9	30 ± 6.7
**PBS**	35 ± 2.8	16.2 ± 2.1	19.6 ± 1.7	18.6 ± 1.5	16.6 ± 2.3	25.8 ± 5.8
**fMLP**	5.5 ± 0.1	5.9 ± 0.2	7.5 ± 0.9	5.5 ± 0.2	8.9 ± 1.7	9.3 ± 3.2
**CD62 negative %**						
**PBS Insert**	10.6 ± 1.8	67.9 ± 6.4	51.1 ± 4.9	36.1 ± 3.9	68.1 ± 9.4	52.3 ± 10.5
**PBS**	48 ± 3.6	79.8 ± 4.6	74.2 ± 3.2	79.2 ± 3.5	75.8 ± 5.0	62.5 ± 8.7
**fMLP**	99,2 ± 0,1	98,7 ± 0,4	94,7 ± 1,9	99,2 ± 0,2	91,9 ± 2,0	90,8 ± 7,6

**Table 2:** Demoscopic data and results of the migration assay of the preterm neonates.

Results (Mean ± SE)	Preterm, vaginal delivery	Preterm, C.S.
**n**	4	18
**Gestational age**	34.6 ± 1.4	34.5 ± 1.8
**Birth weight**	2601 ± 213	2088 ± 128
**Leukocytes/nl**	No data because of small blood sample volume	6.3 ± 0.8
**Number of migrated PMN**		
**PBS**	1525 ± 746	1165 ± 291
**fMLP**	4314 ± 1725	2667 ± 727

We collected anamnestic data concerning the pregnancy and delivery for evaluation resp. exclusion of parameters influencing the neutrophil function, so as maternal medication, drugs like cortisone, antibiotics or tocolysis, laboratory parameters concerning maternal infection, premature rupture of membranes, gestosis or preeclampsia.

To evaluate the influence of the mode of delivery on neutrophil chemotaxis the collective was divided into neonates after vaginal spontaneous delivery (term n = 13, preterm n = 4) and neonates after CS without labor (term n = 31, preterm n = 18). The last group of term neonates was then subdivided regarding the type of anaesthesia into CS under spinal anaesthesia (SA, n = 13), epidural anaesthesia (EDA, n = 11) and general anaesthesia (GA, n = 7). In the group of preterms, all CS were performed under EDA. The standard medication form for the spinal anaesthesia was mepivacaine 4%, 60 mg in combination with sufentanil 5μg i.th. For the EDA 150 mg ropivacaine 0.5% or bupivacaine in combination with sufentanil were given. Induction of GA was done with 25–50 mg ketamine, 3–5 mg/kg thiopental and 1–1.5 mg/kg succinylcholin.

### Sample collection

CB samples taken from the placenta sided umbilical cord immediately after birth and cord clamping. Blood was collected in heparinised blood collection tubes from Sarstedt (Monovette, Lot-Nr.2058507, Nürmbrecht, Germany). A complete blood cell count was obtained from EDTA tubes (Sarstedt) on a hematology analyzer (CellDyn 3500, Abbott, Santa Clara, CA) within 12 hours after blood sampling, stored at 4 – 8°C.

In each series we included blood of a healthy adult volunteer, as a positive assay control and to obtain the reference data for adults (n = 30).

### Flow cytometric assay

The test procedure was carried out according to the manual of the Migratest test kit (Migratest, Item-No. 10-0800, Glycotope, Heidelberg, Germany). In short, Leukocytes were separated within 12 hours after blood sampling by overlaying 1 ml of heparinized whole blood on top of 1ml of Ficoll-solution, left still leading to an upper phase of leukocyte-rich plasma (LRP) after 40 min of spontaneous sedimentation at room temperature without centrifugation. 500 μl of the LRP was aspirated with a transfer pipette and used for migration assay within 1 hour.

350 μl fMLP in a concentration of 10^-6^ M in sterile phosphate buffered saline (PBS) were placed into the wells of a multiwell plate in parallel with PBS as a negative control. Afterwards, cell-culture inserts with 3μm pores in their membranes were placed into these wells, and 100 μL of LRP were added into each insert. Equal amounts of LRP were added into each cell culture insert. Cell concentrations were not adjusted. The grafical correlation analysis of neutrophil counts vs. number of migrated cells either with fMLP or PBS showed no correlation neither in control samples nor in patient samples (data not shown). Therefore, the the number of migrated cells is primarily not dependent of the cell concentration in the insert.

**Fig 1:**
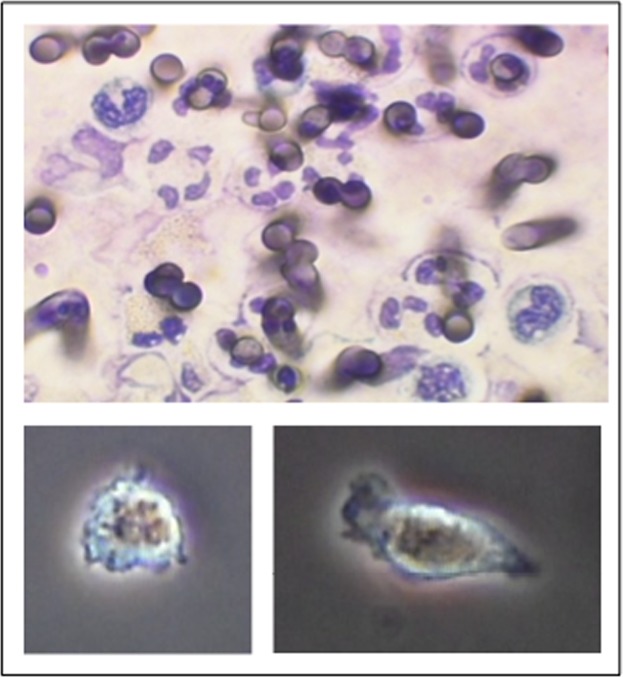
Shape change of PMN during migration through the membrane pores in a fluorescence microscopy analyse Diameter of the pores: 3 μm, see the bevelled, diagonal diameter.

The plates, covered by a lid, were incubated for 30 minutes in a closed water bath at 37°C without shaking. During the incubation period the leukocytes migrated through the pores of the inserts towards the chemotactic agent at the bottom (Fig 1). After incubation time the whole content of the bottom wells was transferred into separate propylene test tubes and stored on ice., Purity of neutrophils was controlled in a fluorescence microscopy analyse. To detach cells possibly attached to the bottom, the wells were gently rinsed using a transfer pipette. When the plate was controlled under an inverted microscope at 200x (Olympus microscope with phase contrast) nothing is left. In addition, 20 μL of the unstimulated, non-migrated cells from the LRP of the PBS-Insert were dispensed in 350 μL of PBS-buffer in a separate test tube and stored on ice. All cell suspensions were labelled with 20 μL of fluorescein isothiocyanate (FITC)-anti-L-Selectin-antibody and counting beads (fluorochrome-conjugated latex micro particles). The tubes were incubated for 10 minutes in the ice bath in the dark. Finally the cells were counterstained with the vital staining DNA dye LDS-751 and incubated for another 5 minutes. All reagents including Ficoll solution and antibodies are supplied as stock solutions in the kit (Migratest, Item-No. 10-0800, Glycotope, Heidelberg, Germany).

Flow cytometry used a FACSCalibur (BD Biosciences) with a 488 nm argon-ion laser light source and Cell-Quest Pro software (BD Biosciences). Fluorescence of LDS-751 (emission maximum at 712 nm) was acquired in FL3-channel (red fluorescence > 630 nm) and green fluorescence of FITC-labelled anti-L-Selectin antibody (emission maximum at 519 nm) in the FL1-channel (530 nm). Red fluorescent neutrophils and counting beads were identified in a SSC vs FL3 dot plot. This gated population in region 1 was displayed in a SSC vs FSC dot plot and the counting beads were gated as region 2. For the purpose of absolute counting of granulocytes data acquisition was stopped when a number of 2000 beads were reached. The intensity of the FSC-signal of neutrophils also reflects the cell size and shape change [25,26]. Instrument performance and stability of fluorescence channels of the FACSCalibur were controlled by daily measurements of Calibrite beads (BD Biosciences).

### Statistical analysis

Statistical comparison between the different patient groups was performed using the nonparametric Wilcoxon signed-rank test for unpaired continuous variables, two-tailed *p* value (WINSTAT, R. Fitch Software, Bad Krozingen, FRG). Results are expressed as mean ± standard error of the mean (SE).

## Results

By the new method we were able for the first time to establish a functional assay of chemotaxis in a neonatal collective out of a small volume of 1 ml blood only without upfront manipulation of the fragile neutrophils. In a set up phase it was assured that the method yielded good reproducible results in peripheral blood from adults as well in CB of term neonates, even in CB of preterm neonates including a stillborn fetus with a gestational age of 22 weeks (data not shown).

We first set up a reference range for neutrophil chemotaxis in CB of healthy term neonates born by vaginal delivery and then compared it with blood of healthy adults and then in a next step with those of preterm neonates

### Comparison of neutrophil chemotaxis in cord blood of healthy term neonates after vaginal delivery and healthy adults

Mean leukocyte counts (WBC) and neutrophil counts out of the complete blood count (CBC) are shown in Table 1 as well as the functional assay results.

#### Neutrophil migration

**Fig 2:**
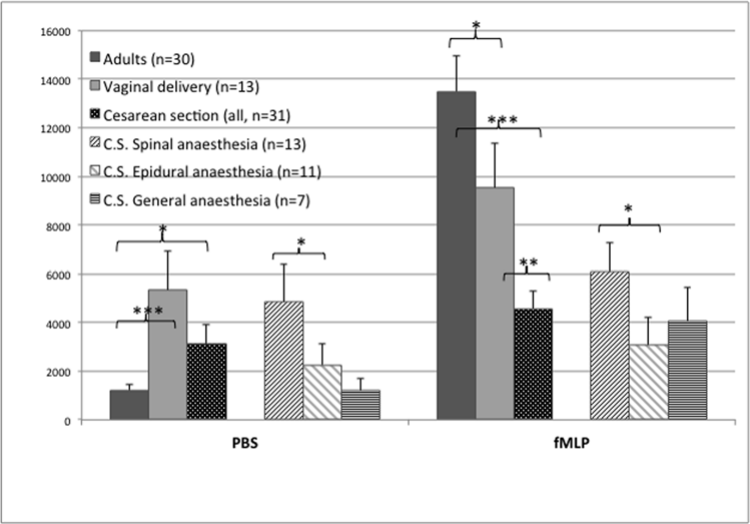
Neutrophil migration in adults (n = 30) and in cord blood of healthy term neonates expressed as number of migrated cells on the ordinate and the tested chemoattractants (PBS-buffer and fMLP) on the abscissa. Neonates after vaginal delivery (n = 13), Cesarean section (CS, all, n = 31), CS under spinal anaesthesia (n = 13), CS under epidural anaesthesia (n = 11), CS under general anaesthesia (n = 7). Results are expressed as Mean ± SE. * p<0.05; ** p<0.01; *** p<0.001

The random migration towards PBS (chemokinesis) was significantly higher (p = 0.008) in CB neutrophils than in adults (see Fig 2), but neutrophil migration towards fMLP (chemotaxis) could be stimulated to a higher level in adult cells (p = 0.02). To elucidate this finding we expressed this degree of stimulation as a ratio of cells migrated to fMLP versus migration to PBS, which is a factor of 1.8 for CB but 11 for the adult cells, a first striking difference we found between healthy newborns and adults.

The independent parameter of shape change of neutrophils, a prerequisite for the active migration, confirmed the findings of the cellular migration. CB neutrophils were significantly larger than the adult cells before stimulation (cell size measured as mean forward scatter signal), but adult cells showed a higher enlargement after stimulation with fMLP (see Table 1).

#### Expression of L-Selectin on the neutrophil surface

**Fig 3:**
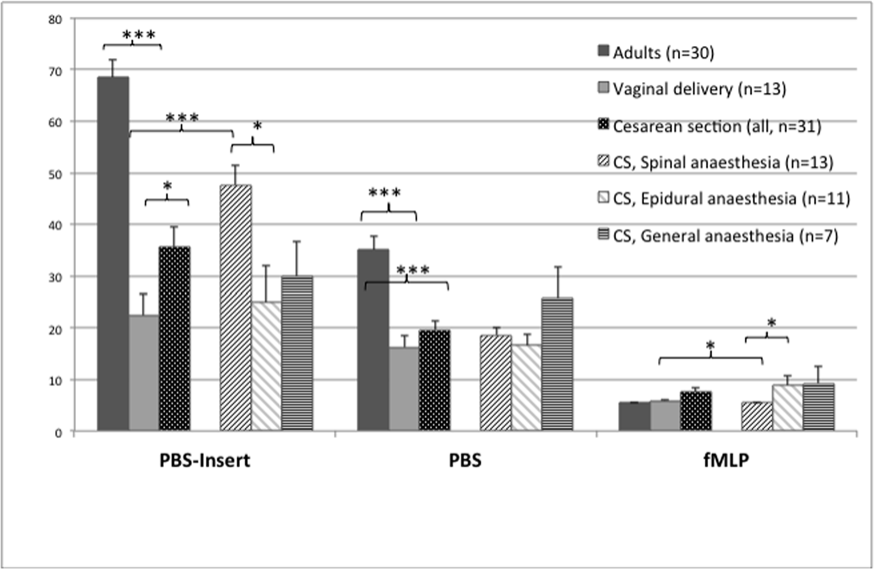
L-Selectin expression on neutrophils granulocytes in adults (n = 30) and in cord blood of healthy term neonates expressed as relative fluorescence intensity (RFI) on the ordinate and the tested cell populations on the abscissa: non-migrated cells from PBS-insert (left) and migrated cells towards PBS-buffer (middle) and fMLP (right). Neonates after vaginal delivery (n = 13), Cesarean section (CS, all, n = 31), CS under spinal anaesthesia (n = 13), CS under epidural anaesthesia (n = 11), CS under general anaesthesia (n = 7). Results are expressed as Mean ± SE. * p<0.05; ** p<0.01; *** p<0.001

L-Selectin expression on the neutrophil surface, displayed as the Mean of the RFI of the fluorescent antibody, was analysed in the cell suspension after migration through the membranes towards PBS and fMLP as well as in the remaining LRP of the PBS-insert, which served as a control before start of migration (see Fig 3). Even before migration through the membrane pores the L-Selectin expression on the neutrophil surface was significantly lower (p < 0.001) on CB cells. Random migration towards PBS resulted in a further loss of L-Selectin, whereas chemotaxis towards fMLP produced a maximal shedding of L-Selectin in both groups. Given the expression in the PBS-insert as 100%, the expression was reduced by fMLP to a level of 30% positivity in CB and down to only 8% in adult cells.

### Comparison of neutrophil chemotaxis in cord blood of vaginally born term neonates with newborns after Cesarian section without labor

Mean leukocyte counts were 12,6 / nL for VB and 9,2 / nL for CS (p = 0.02).

#### Neutrophil migration

CB PMN of neonates after CS showed significantly less random migration (chemokinesis) and less fMLP-stimulated migration (chemotaxis) compared to PMN from vaginally born (VB) infants (p = 0.005). This difference in activation rate of PMN was also observed in the change of the cell size, which rises as a result of shape change when neutrophils are stimulated. FSC was significantly lower in the PMN of CS neonates compared to VB. The differences were highly significant for non-migrated cells as well as for PMN after random migration and stimulation with fMLP (all p < 0.001, see Table 1). These results of cellular migration and shape change in CB complement each other in the sense of higher neutrophil activation after VB compared to CS.

#### Expression of L-Selectin on the neutrophil surface

Levels of L-Selectin on CB PMN before migration were significantly higher, meaning less reduced in CS compared to VB neonates (p = 0.03). After neutrophil migration (random and stimulated), L-Selectin was down-regulated in both groups and there was no significant difference between VB and CS neonates.

### Neutrophil chemotaxis of neonates after Cesarean Section under different types of anaesthesia

In order to elucidate a possible effect of anaesthesia, the neonates after CS (n = 31) were subdivided into 3 groups: CS under SA, EDA and GA. There was no significant difference in mean gestational age and birth weight between these groups (see Table 1).

Neonates after CS under EDA and GA showed a reduced chemokinesis (p = 0.07 and 0.03, resp.) and less chemotactic response (p = 0.004 and 0.02, resp.) compared to VB neonates.

In case of CS under SA, random and fMLP-stimulated PMN migration were only slightly reduced compared to VB (not significant) and were significantly higher than after CS under EDA (p = 0.047 for random and 0.01 for fMLP-stimulated migration) and GA (see Fig 2 and Table 1).

Neutrophil cell size from neonates after VB was significantly higher than after CS under SA, EDA and GA. Elevation of cell size seems to be a marker for pre-activation of PMN after VB, which could be caused by the physiological process of labor. We found the lowest neutrophil cell size in the EDA group, meaning the lowest activation rate in all assays, an observation that matches with the findings of lowest random and stimulated PMN migration in EDA cases (Table 1).

L-Selectin expression on non-migrated neutrophils reached the highest values (lowest pre-stimulation) in neonates after SA and was significantly higher than after VB (p = 0.0002) and after CS under EDA (p = 0.01) and GA. After stimulation with fMLP, we found down-regulation of L-Selectin in all groups (Fig 3). The lowest L-Selectin expression was found on fMLP-stimulated neutrophils after VB and after CS under SA, which were significantly lower than after EDA (p = 0.03) and GA.

Looking at the difference between baseline L-Selectin-expression before stimulation and after stimulation, neutrophils of SA infants shed the highest amount of L-Selectin of all groups. When being stimulated with fMLP, the PMN from neonates after EDA and GA showed less L-selectin-shedding, which means less reactivity upon activation.

### Neutrophil chemotaxis in cord blood of preterm neonates

In preterm neonates < 37 weeks of gestation (n = 22, for population see Table 2), we had to subdivide the population again into VB and CS as we were able to show an influence of the mode of delivery and anesthesia. The number of VB preterms was quite small in that population, because in most cases a CS was clinically indicated. All CS were performed under EDA. First, the differences depending on the mode of delivery were confirmed for preterm neonates, as neutrophils after VB show a higher migration rate.

**Fig 4:**
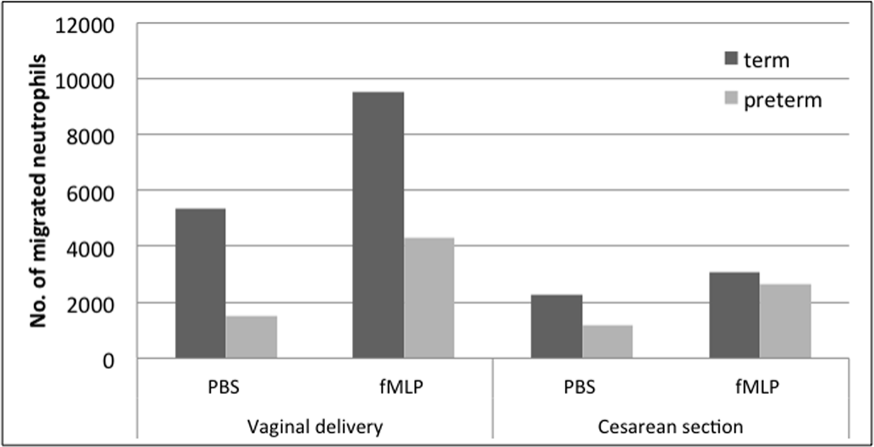
Neutrophil migration in cord blood of term and preterm neonates expressed as number of migrated cells on the ordinate and the tested chemoattractants (PBS-buffer and fMLP) and patient populations on the abscissa. Left: Term (n = 13) and preterm (n = 4) infants after vaginal delivery Right: Term (n = 11) and preterm (n = 18) infants after Cesarean section under epidural anaesthesia. Results are expressed as Mean ± SE. No statistically rsignificant differences.

The reduction in neutrophil migration in VB preterm neonates especially in the spontaneous migration (p = 0.2, Fig 4) was not significant because of the small number of VB preterms.

CS under EDA nearly showed the same level of reduced neutrophil migration in term and preterm neonates. However, in preterm neonates the neutrophils were not impaired in their ability to change their cellular shape (data not shown).

The high L-Selectin expression on un-stimulated and stimulated PMN showed no statistical difference between term and preterm neonates (data not shown). Taken together, the shape change and L-Selectin-expression and shedding should not be responsible for the reduced neutrophil migration in preterm neonates.

## Discussion

We were able to show by a new and robust flow cytometric assay on small sample volumes that in CB of healthy term neonates after VB chemokinesis was significantly higher than in adults. The ability for chemotaxis was higher in adults what is confirmatory to several published results [1,2]. The absence of neutrophil damage in our assay avoiding neutrophil isolation could explain the higher rate of fMLP-activated CB neutrophils in our study and puts older data from Krause [13] in question who showed a fraction of poorly motile PMN. The significantly lower L-Selectin expression on CB neutrophils represents a pre-activation and fits well to the observed elevated spontaneous migration. Chemotaxis and chemokinesis both trigger a more or less marked shedding of L-Selectin in both groups, more effective in the adults.

Referring to other studies which showed that the mode of delivery does not affect the chemotaxis of CB neutrophils like the one from Herson [27]. The group from of Fox [5] analysed 40 mL of CB only after elective CS, and needed to manipulate the blood samples by centrifugation. We also confirm the findings of a significantly reduced neutrophil migration in CB after CS without labor as described by other publications.

In our investigations the neutrophils from adults could be stimulated to a higher rate of migration compared to the CB cells. As neonates do not seem to have severe deficiencies in their responsiveness to the important neutrophil chemoattractants – Fox et al. showed that neutrophils from CB exhibit the same pattern of potency for several chemokines like adult neutrophils – other reasons for the higher activation by fMLP of adult neutrophils are discussed.

### Clinical aspects of Neutrophil chemotaxis in neonates

CB PMN of neonates after CS showed less chemokinesis and less migration after stimulation with fMLP compared to PMN of VB infants. We cannot rule out the influence of labor alone because we had no cases of CS done after labor but, neonates after CS, independent of EDA or GA showed less PBS- and fMLP-induced migration compared to VB neonates and to those after CS under SA, which was only slightly reduced compared to VB. The highest L-Selectin-shedding was found on fMLP-stimulated neutrophils after VB and after SA, which were significantly lower after EDA and GA. So today’s anesthetic standard regime of spinal anesthesia (SA) showed the least influence on the neutrophil migration in CB. Intrathecal application of the local anesthetic drug in SA has the advantage that lower doses are needed and lower concentration of the drug passes the placenta to the neonate [14]. Gasparoni observed reduced migration of neutrophils derived from CB after CS under EDA than after VB with a significant inverse relationship between chemotaxis and lidocaine levels. He used a microscopical measurement of the distance of the leading neutrophils of the zone of migrating cells. Sasagawa [23] examined in vitro the effect of tetracaine, lidocaine, prilocaine and procaine on the chemotaxis after fMLP-stimulation. He found a dose dependent inhibitory effect on the PMN-chemotaxis also related to the anesthetic potency. Mikava [24] investigated the effect of ropivacaine, bupivacaine and lidocaine on the neutrophil chemotaxis, phagocytosis, burst, intracellular Ca 2+ ion concentration and protein kinase C activity in vitro in plasma concentrations that are used clinically. Neither ropivacaine nor bupivacaine, used for EDA in our patients, impaired the neutrophil chemotaxis in vitro. Fischer [21] studied the influence of lidocaine, tetracaine and ropivacaine on the lysophosphatidic acid (LPA) signaling for neutrophil chemotaxis and respiratory burst in vitro. The chemoattractive effect of LPA was attenuated only by lidocaine.

These in vitro studies are in accordance with the reduced neutrophil chemotaxis in CB found in patients after CS under EDA with lidocaine as described by Gasparoni et al. [22]. Those in vitro studies referred to above found no influence of bupivacaine or ropivacaine on the neutrophil migration. Nevertheless our ex-vivo observations showed significantly reduced chemokinesis and chemotaxis in CB after epidural anesthesia with both of these local anesthetics, whereas the neutrophil migration after CS under spinal anaesthesia with mepivacaine was not reduced compared to VB neonates.

We saw a difference between the term and preterm neonates with a tendency of less neutrophil migration in preterm neonates, especially in the spontaneous migration without any significant statistical difference. But we emphasize that the neutrophils of the preterm neonates were able to react on the fMLP-stimulus and showed an increased chemotactic response comparable to the term neonates proceeding from the lower spontaneous migration. Furthermore the neutrophils of preterm neonates have no deficit in the ability of changing the cellular shape. So, shape change should not be the responsible factor for the decreased neutrophil migration in preterm neonates.

Usmani et al. [19] evaluated the random chemotactic motility in preterm neonates younger than 32 wks of gestation. Their first measurement was on day 10 after birth, where the neutrophils of the preterm neonates showed significantly less random migration than those of the term neonates. Already after three weeks of life, the random motility in term and preterm neonates was comparable. Zentay et al. [28] described the decreased neutrophil chemotaxis in CB using dexamethasone in vitro caused by the inhibition of the PMN´s proper IL-8 release and the study group of Fuenfer et al. [7] showed a significant reduced neutrophil chemotaxis and random migration in CB using betamethasone in a micropore filter assay in a concentration found in CB following a standard dose administered to the mother. Gasparoni [14] showed that there was no significant correlation between chemotaxis and endogenous cortisol level in CB. So the influence of the clinically indicated infant respiratory distress syndrome (IRDS)-prophylaxis to the pregnant women in a part of our study patients on the neutrophil migration is not finally clear. A larger study population with defined groups would be necessary.

## Conclusion

The new flow cytometric assay of neutrophil chemotaxis is an appropriate and objective method to analyse functional differences even in very small volumes of blood, essential in neonatology. Term neonates do not show reduced chemotaxis compared to adults; contradictory findings of other investigators did not take into consideration the different modes of delivery and anaesthesia. Preterm neonates present with reduced chemotaxis and chemokinesis, confirming the well known deficits in their neutrophil function. The side effects of maternal drugs on the neonatal immune system have to be considered especially when the immune response is already impaired, as in preterm infants.
